# Technology, innovation and health equity

**DOI:** 10.2471/BLT.15.155952

**Published:** 2015-07-01

**Authors:** Hildy Fong, Eva Harris

**Affiliations:** a Center for Global Public Health and Division of Infectious Diseases and Vaccinology, School of Public Health, University of California, Berkeley, CA, United States of America.

Innovative technologies have enormous potential to improve human well-being. However, technological progress does not guarantee equitable health outcomes. As advances in technology redefine the ways people, systems and information interact, resource-poor communities are often excluded. Where technological fixes have been imposed on communities, the results have included abandoned equipment, incompatible computer programs and ineffective policies.

A shift in values among leadership, communities and the creators of technology is critical to implementing technology sustainably and equitably. Numerous examples exist of technological applications that undermine equity, fairness and human rights: for example, the use of high-tech medical interventions in preference to simpler preventive measures or terminator genes that prevent the re-use of seeds for food crops. To ensure equitable outcomes, the design and implementation of technology need to respect ethical principles and local values. Decisions on the use of new technology should be made by local users, and implementation needs long-term commitment and local ownership. Here, we discuss features of technology implementation that can promote health equity, using a range of examples from the health, agriculture and economic sectors.

Successful examples of technological implementation illustrate the core values of equity. The Sustainable Sciences Institute (SSI) helps develop and implement technologies, in partnership with local communities, to combat infectious diseases in low-income settings. SSI’s approach involves community-centred capacity building including training programmes, small grants, material aid and partnerships to provide long-term support.^1^^,^^2^ Laboratory techniques such as reverse transcription polymerase chain reaction (RT–PCR), enzyme linked immunosorbent assay, cell culture and flow cytometry have been applied in resource-poor settings by adapting these technologies on-site and strengthening local knowledge of the principles and limitations of each technique.^1^

In Nicaragua, scientists can now develop diagnostic kits locally for diseases including dengue, leptospirosis and American trypanosomiasis, and diagnosis can be done by regional as well as central laboratories. Real-time RT–PCR testing for pandemic influenza was operational before the first case presented in the 2009 pandemic. The Nicaraguan National Virology Laboratory recently developed and implemented diagnostic tests for Chikungunya, a mosquito-borne viral disease that was recently introduced into Central America and the Caribbean. Training local scientists, reducing scientific isolation and promoting international collaboration enables rapid, local response to communicable disease outbreaks.

Related examples exist worldwide in the arena of information and communication technologies for health. Infectious disease surveillance and laboratory and clinical management can be improved using low-cost information systems, but these must be based on local human resource capacity, hardware and software availability, and connectivity.^1^^,^^3^ SSI partnered with the Nicaraguan Ministry of Health to design information systems to improve data quality, reduce costs and increase decision-support for multiple end-users. In this context, mobile (mHealth) tools and web-based systems for tracking paediatric immunization, prenatal health and community health data have improved access to information at the primary care level.^1^ Hesperian Health Guide’s Digital Commons project shows how health information can be accessible to millions of users in digital and multi-media formats.^4^ The content – developed and refined with partner communities worldwide – is freely available via the internet and mobile tools, increasing equitable access to critical resources at the community level.

Nongovernmental organizations such as BRAC (formerly the Bangladesh Rural Advancement Committee) support impoverished communities by using innovation to leverage their own human and material resources. In the 1960s, oral rehydration therapy was identified as a ground-breaking yet simple way to treat diarrhoeal diseases. However, it was not until oral rehydration was integrated into community health strategies that its impact was fully realized.^5^ BRAC trained 13 million rural women in Bangladesh to treat children in their communities using simple tools to measure salt-sugar-water solution ratios.^5^

In the agricultural arena, organizations such as La Via Campesina reach hundreds of thousands of farmers by teaching agro-ecological principles, improving yields while conserving natural resources and biodiversity. Capacity building that empowers communities can promote food sovereignty and mitigate determinants that perpetuate hunger. Diversified farming systems protect ecosystems by increasing the genetic diversity of crop varieties and livestock. Biodiverse and ecologically sustainable approaches are proving more productive and resilient to changing environmental conditions than modern monoculture.^6^

Alternative economic models that espouse the core principles of equity include microfinancing and grassroots entrepreneurship. Programmes like BRAC, Grameen and Ashoka mobilize microfinance as a social platform to deliver scaled-up services in health, education, business development and livelihood support – all critical components in breaking the cycle of poverty. Thus, technology can promote health equity, if implemented in partnership with communities and based on core values of local autonomy, fairness and ecological sustainability.
